# Malignant Pleural Effusion: Palliative Managements and Indication for Pleurodesis Based on Survival Scores

**DOI:** 10.1002/jso.28156

**Published:** 2025-05-20

**Authors:** Erlon de Avila Carvalho, Rachid Eduardo Noleto da Nobrega Oliveira, José Henrique Agner Ribeiro, Jefferson Luís Gross, Cezar Augusto Vendas Galhardo, Heládio Feitosa e Castro Neto, Giovana dos Santos, Reitan Ribeiro, Alexandre Ferreira Oliveira, Rodrigo Nascimento Pinheiro

**Affiliations:** ^1^ Brazilian Society of Surgical Oncology Rio de Janeiro Brazil; ^2^ Thoracic Surgery and Pneumology Service, Hospital das Clínicas of Federal University of Minas Gerais, Mário Penna Institute Belo Horizonte Minas Gerais Brazil; ^3^ Thoracic Surgery Service, Barretos Cancer Hospital São Paulo São Paulo Brazil; ^4^ Thoracic Surgery Service, AC Camargo Cancer Center São Paulo São Paulo Brazil; ^5^ Thoracic Surgery Service, Alfredo Abrão Cancer Hospital Campo Grande Mato Grosso do Sul Brazil; ^6^ Surgical Oncology Service, Cancer Institute of Ceará Fortaleza Ceará Brazil; ^7^ Surgical Oncology Service, Erasto Gaertner Hospital Curitiba Paraná Brazil; ^8^ Surgical Oncology Service Federal University of Juiz de Fora Juiz de Fora Minas Gerais Brazil; ^9^ Surgical Oncology Department Hospital de Base of the District Federal Brasília District Federal Brazil

**Keywords:** guidelines, malignant pleural effusion, palliative care, pleurodesis, survival scores, thoracic surgery

## Abstract

Neoplastic pleural effusion is one in which malignant neoplastic cells are detected in the pleural fluid or in the parietal pleura. When secondary, it confirms disseminated neoplastic disease and suggests a reduced life expectancy and low quality of life. This review was described by a group of physician members of the Brazilian Society of Oncological Surgery regarding the treatment of neoplastic pleural effusion, developed to guide surgeons, palliative care physicians and clinical oncologists in their clinical practice to assess patients indicated for pleurodesis based on survival scores.

## 1 Introduction

Neoplastic pleural effusion, by definition, is one in which malignant neoplastic cells are detected in the pleural fluid or in the parietal pleura. When secondary, it confirms disseminated neoplastic disease and suggests a reduced life expectancy and low quality of life. Most cases are secondary to pleural metastases from breast and lung cancer (50%–65%), and the average survival ranges from 3 to 12 months depending on the primary tumor and histological type, being shorter in cases of lung cancer and longer in cases of ovarian and breast cancer. About 10% are primary, usually due to pleural mesothelioma [1, 2].

Accurate survival prediction is vital for planning effective palliative care, whether to indicate a surgical approach or some clinical treatment. To determine how appropriate a medical intervention is for a patient, physicians need to estimate the risks and benefits of each treatment based on their projected prognosis [3]. Survival scores in oncology, especially in the context of malignant pleural effusions (MPE), are crucial tools for risk stratification and treatment planning. The Palliative Prognostic Index (PPI) is a prognostic score used to estimate survival in patients with advanced cancer, particularly in palliative care settings [4]. The LENT score, which considers LDH in pleural fluid, Eastern Cooperative Oncology Group (ECOG) performance status, the neutrophil–lymphocyte ratio, and tumor type, is one of the main scores used to predict survival in patients with MPE [4].

A prospectively validated score (PROMISE) published in 2018 predicted survival better than the LENT score. This was the first prognostic score to combine biological markers and clinical parameters. The PROMISE score includes seven variables (chemotherapy, radiotherapy, hemoglobin, white blood cell count, C‐reactive protein (CRP), ECOG, and cancer type), in addition to the tissue inhibitor of metalloproteinases 1 (TIMP1) for the biological score [5].

Pleural and oncological treatment options are expanding, and therefore a more accurate prognosis at presentation can help individualize treatment strategies [6, 7]. Treatments can cause morbidity and require hospitalization; thus, identifying patients with the worst prognosis can help minimize discomfort and inconvenience at the end of their lives.

This review was described by a group of physician members of the Brazilian Society of Oncological Surgery regarding the treatment of neoplastic pleural effusion, developed to guide surgeons, palliative care physicians, and clinical oncologists in their clinical practice to assess patients indicated for pleurodesis based on survival scores.

## 2 Epidemiology

The global incidence of cancer continues to rise, with a projected increase of about 66% between 2012 and 2030 [8]. This can be explained by the increase in life expectancy associated with new treatments for other diseases and improved lifestyle habits. MPE is a common disease in clinical practice, with an annual incidence of 660 new cases per million inhabitants, resulting in more than 1 million people affected globally each year [9, 10]. It typically reflects an advanced stage of disease, with a worsening quality of life and reduced life expectancy, with global survival after diagnosis varying from 3 to 12 months [11, 12].

Malignant neoplasia is one of the most common causes of pleural effusion. Approximately 20% of all pleural effusions are malignant, and 15% of all patients with neoplasms will develop MPE during their disease progression [13]. If we consider only patients with metastatic neoplasms, this number rises to 50% [14]. Most cases are secondary to lung and breast cancer, together accounting for 50%–65% of all MPE cases [6]. Tumors of the gastrointestinal tract and lymphomas account for ~25% of cases, and about 10% of cases remain with an unknown primary site [15]. In primary MPE cases, 90% of pleural mesothelioma cases, and 5%–33% of primary pleural lymphoma cases are related to MPE [16, 17]. There are also rare cases documented in the literature of thyroid neoplasms, multiple myeloma, angiosarcoma, synovial sarcomas, and others.

Cases of MPE secondary to ovarian and breast cancer generally have a longer survival than those of lung cancer, where the 5‐year survival rate is only 3% [18]. In the USA, MPE cases are individually responsible for more than 360 000 hospital admissions/year, with a mortality rate of ~12%, and a cost of 10 billion dollars/year [19, 20]. In Brazil, excluding nonmelanoma skin cancer, there will be about 480 000 new cancer cases/year from 2023 to 2025. Considering this statistic, there will be about 72 000 MPE cases in 2025, with breast and lung cancer accounting for approximately 36–46 thousand of these [21]. Consequently, MPE has a substantial impact on the healthcare system due to its high readmission rates and significant resource utilization [20].

## 3 Pathophysiology

MPE is a common complication in advanced malignancies, particularly associated with lung, breast, and ovarian cancers [22]. Its pathophysiology involves complex processes mediated by interactions between tumor cells, the pleural microenvironment, and components of the immune system [23]. Tumor cells invading the pleural space produce cytokines and growth factors, such as vascular endothelial growth factor (VEGF), which plays a central role in MPE formation [24]. VEGF increases vascular permeability by promoting transcellular gap formation and destabilizing intercellular junctions, leading to the extravasation of protein‐rich fluid into the pleural space [24, 25].

In addition to VEGF, other mediators, such as secreted phosphoprotein‐1 (SPP1), contribute to MPE development [26]. SPP1, secreted by both tumor and host cells, promotes inflammation, angiogenesis, and the recruitment of inflammatory cells, such as macrophages and mast cells, upon recruitment to the pleural space, mast cells degranulate and release inflammatory mediators that exacerbate vascular permeability [27]. Experimental models have demonstrated that the absence of mast cells or the blockade of SPP1 and VEGF significantly reduces MPE volume, indicating these mediators as potential therapeutic targets [28].

Inflammation in MPE is further amplified by specific immune cells, such as Th17 lymphocytes, which exhibit dual roles [29]. Although these lymphocytes are known for their pro‐inflammatory functions, studies suggest that in certain contexts, they can negatively regulate MPE progression [30]. Cytokines produced by Th17 cells, including IL‐17 and IL‐22, are elevated in MPE and contribute to tumor microenvironment remodeling and interactions between tumor and immune cells [31]. Cross‐signaling among Th17 lymphocytes, macrophages, and regulatory T cells highlights the complexity of the malignant pleural microenvironment [32].

The development of MPE is also associated with mechanical obstruction of pleural lymphatic and blood vessels, impeding fluid drainage [33, 34]. Additionally, the presence of tumor cells in the pleural space results in the release of proteases, such as matrix metalloproteinases, which degrade pleural tissue, facilitating tumor invasion and perpetuating a vicious cycle of inflammation, angiogenesis, and fluid accumulation [28, 35]. Thus, therapeutic interventions targeting these key mediators may provide symptomatic relief and potentially benefit disease progression.

## 4 Diagnosis

The diagnosis of MPE requires an integrated approach involving imaging techniques, pleural fluid analysis, and, when necessary, pleural biopsies. These steps are critical to confirming malignancy and guiding clinical management.

1. Imaging Techniques

- Chest X‐Ray: Often the initial examination, useful for identifying the presence and laterality of pleural effusion, while widely available, its sensitivity for detecting small fluid volumes or malignant pleural changes is limited [36].
- Computed Tomography (CT): Regarded as the gold standard for detailed pleural space assessment, CT can identify features suggestive of malignancy, such as pleural thickening, nodularity, and mediastinal involvement [37].
- Thoracic Ultrasound: In addition to detecting small fluid volumes, it facilitates procedures such as thoracentesis, features like irregular pleural thickening or nodularity may indicate malignancy [37, 38].

2. Pleural Fluid Analysis

- Thoracentesis: This initial procedure collects pleural fluid for analysis [39]. Cytological evaluation detects malignant cells in ~60% of MPE cases [39, 40]. Furthermore, biochemical tests such as protein levels, LDH, and pH help distinguish exudates from transudates [41].
- Light's Criteria: Essential for classifying pleural fluid as exudate or transudate the criteria include a ratio of pleural fluid protein to serum protein > 0.5 a ratio of pleural fluid LDH to serum LDH > 0.6 and pleural fluid LDH greater than two‐thirds of the upper limit of normal serum LDH while effective Light's criteria may overestimate exudates in patients using diuretics in such cases the serum‐pleural fluid albumin gradient can refine the diagnosis [42, 43, 44].

3. Biopsy Methods

The diagnostic approach for MPE often includes image‐guided pleural biopsy and medical thoracoscopy, particularly when initial cytology is inconclusive [45]. Image‐guided pleural biopsy, such as those performed using CT or ultrasound, is recommended when imaging reveals pleural thickening or nodules [38]. This technique allows for targeted sampling of pleural lesions, thereby enhancing diagnostic accuracy in cases where cytology alone is insufficient [41].

Medical thoracoscopy, also known as pleuroscopy, is a minimally invasive procedure that enables direct visualization of the pleural space and facilitates multiple biopsies from different sites [46]. This method is especially valuable in cases of negative or inconclusive pleural fluid cytology, with a diagnostic yield exceeding 95% [41]. In addition to its effectiveness in diagnosing conditions such as malignant pleural mesothelioma, medical thoracoscopy can be performed under local anesthesia or moderate sedation, often in an outpatient setting [47, 48]. The procedure also allows for therapeutic interventions, such as pleurodesis [49].

The choice between image‐guided biopsy and medical thoracoscopy depends on factors such as the presence of pleural thickening or nodules, the patient's clinical condition, and the availability of resources and expertise [50]. While image‐guided biopsy is less invasive and may suffice in certain cases, medical thoracoscopy provides a higher diagnostic yield and is preferred when a more comprehensive evaluation of the pleural space is required [45, 51].

These diagnostic strategies are indispensable for confirming MPE, characterizing its extent, and planning treatment, focusing on palliation and symptom control.

## 5 Treatment

The primary goal of therapeutic management should be symptom relief, with an emphasis on improving quality of life. Typically, there is no survival benefit, making this a palliative treatment. Therefore, the focus should be on less aggressive, less invasive, and more economically viable treatments, preferably outpatient‐based or provided in a day hospital setting.

To determine the ideal treatment, it is first necessary to assess the patient's clinical conditions, the type of treatment they are currently receiving, and, most importantly, their estimated survival. The treatment is invasive, uncomfortable, and impacts quality of life. A better understanding of survival expectations can help guide treatment decisions.

Survival scores in oncology, particularly in the context of MPE, are crucial tools for risk stratification and treatment planning. The use of prognostic scores to estimate survival in cancer patients has long been established, with examples such as the PPI [3]. This score is a prognostic tool used to estimate survival in patients with advanced cancer, particularly in palliative care settings. It incorporates clinical parameters such as performance status, oral intake, edema, resting dyspnea, and delirium to stratify patients into different prognostic categories. The PPI is particularly useful for predicting short‐term survival, with scores > 6 indicating a survival of < 3 weeks and scores > 4 indicating a survival of < 6 weeks [52, 53] (Table 1). Its ease of use and independence from invasive tests make it suitable for various care settings, including hospitals and home care [53].

**Table 1:** Palliative Prognostic Index—PPI.

Factor	Score	Total score	Survival prediction
PPS 10%–20%	4.0	Score < 4.0	> 6 weeks
PPS 30%–50%	2.5	Score > 4.0	< 6 weeks
PPS > 50%	0	Score > 6.0	< 3 weeks
Delirium	4.0		
Dyspnea at rest	3.5		
Severely reduced oral intake	2.5		
Reduced oral intake	1.0		
Normal intake	0		
Edema	1.0		

*Note:* Adapted from PPI, Morita et al. [3].

When a PPI score > 6 was used as a cutoff point, a 3‐week survival was predicted with a sensitivity of 80% and a specificity of 85%. When a PPI score > 4 was used as a cutoff point, a 6‐week survival was predicted with a sensitivity of 80% and a specificity of 77% [3]. Based on the PPI, we suggest the indication and contraindication for pleurodesis (Table 2).

**Table 2:** Association of PPI score with indication for pleurodesis.

Scores	Survival	Indication for pleurodesis
< 4.0	> 6 weeks	Yes
> 4.0	< 6 weeks	Evaluate case by case
> 6.0	< 3 weeks	No

Recently, the use of survival scores has been proposed to estimate the survival of MPE, such as the LENT score and PROMISE, which help guide treatment decisions and predict the clinical evolution of patients. The LENT score is a prognostic tool developed to predict survival in patients with MPE. It incorporates four main parameters: pleural fluid lactate dehydrogenase (LDH), ECOG performance status, neutrophil‐to‐lymphocyte ratio, and tumor type. Each of these components receives a score, and the total LENT score categorizes patients into low, moderate, or high‐risk mortality groups (Table 3).

**Table 3:** LENT score for prognosis of malignant pleural effusion.

Points				
	0	1	2	3
Pleural fluid LDH, U/L	< 1.500	≥ 1.500	—	—
ECOG score	0 (asymptomatic)	1 (symptomatic, but ambulatory)	2 (symptomatic, in bed < 50% of the day)	3–4 (symptomatic, in bed > 50% of the day or bedridden)
Serum neutrophil‐to‐lymphocyte ratio	< 9	≥ 9	—	—
Tumor type	Mesothelioma or hematologic malignancy	Breast, gynecological cancer, or RCC	Lung cancer or any other cancer	—

Score	Risk	Median survival
0–1	Low	319 days
2–4	Moderate	130 days
5–7	High	44 days

The LENT score has been validated and effectively stratifies patients based on their survival prospects. In the original study, patients were classified into low, moderate, and high‐risk groups, with median survival times of 319, 130, and 44 days, respectively [54]. The score demonstrated superior predictive accuracy compared to ECOG performance status alone, particularly at 1, 3, and 6 months after diagnosis [54].

However, the LENT score may not be universally applicable to all patient populations. For example, in patients with MPE secondary to lung adenocarcinoma, particularly those with EGFR mutations, the LENT score may underestimate survival, suggesting the need for potential modifications in these subgroups [55]. Moreover, studies have explored the integration of other prognostic factors, such as EGFR mutation status, to refine the predictive ability of the LENT score in specific populations, such as Asian patients [55].

Overall, the LENT score is a valuable tool for guiding clinical decision‐making in MPE, providing a structured approach to estimating patient prognosis and tailoring treatment strategies accordingly.

However, we must emphasize that patients with EGFR mutations and ALK translocation who receive tyrosine kinase inhibitors have different outcomes compared to those undergoing conventional chemotherapy [56, 57] (Table 4).

**Table 4:** Comparison of the mean survival based on the LENT score in patients receiving targeted therapy with tyrosine kinase inhibitors or conventional chemotherapy [56].

Risk category (LENT score)	Patients receiving target therapy	Patients receiving conventional chemotherapy	*p*
Survival days, median (IQR)			
High risk (LENT score > 5)	238 (27–not available)	63 (11–77)	*p* < 0.05
Moderate risk (LENT score 2–4)	1033 (245–1710)	109 (31–406)	*p* < 0.05
Low risk (LENT score 0–1)	—	—	***—***
Survival rate at 6 months			
High risk (LENT score > 5)	89%	45%	*p* < 0.05
Moderate risk (LENT score 2–4)	67%	10%	*p* < 0.05
Low risk (LENT score 0–1)	—	—	*—*

Based on this, when calculating survival scores, we should consider whether patients are receiving tyrosine kinase inhibitors. To date, no study has compared the use of immunotherapy with survival scores in MPE.

The PROMISE score, published in 2018, demonstrated better survival prediction than the LENT score. It was the first prognostic score to combine biological markers and clinical parameters, incorporating seven variables (chemotherapy, radiotherapy, hemoglobin, white blood cell count, CRP, ECOG performance status, and cancer type), along with pleural fluid TIMP1 for the biological PROMISE score [5] (Table 5).

**Table 5:** PROMISE score with its variables.

		Points	
Variable		PROMISE clinical score	PROMISE biological score (with TIMP1)
Previous chemotherapy	No	0	0
	Yes	4	3
Previous radiotherapy	No	0	0
	Yes	2	2
Hemoglobin, g/dL	> 16	0	0
	14 to 16	1	1
	12 to 14	2	2
	10 to 12	3	3
	< 10	4	4
Serum leukocyte count, 10^9^ cells/L	< 4	0	0
	4 to 6.3	2	2
	6.3 to 10	4	4
	10 to 15.8	7	7
	> 15.8	10	9
C‐reactive protein, IU/L	< 3	0	0
	3 to 10	3	3
	10 to 32	5	5
	32 to 100	8	8
	> 100	11	10
ECOG performance status	0–1	0	0
	2–4	7	7
Cancer type	Mesothelioma	0	0
	All other cancer types	4	5
	Lung	5	6
TIMP1, ng/mg of protein	< 40	__	0
	40 to 160	__	1
	> 160	__	2

Although the PROMISE score estimates survival more accurately, the measurement of TIMP1 is not always available in hospitals and laboratories. Therefore, we opted to use the LENT score in combination with the clinical parameters of the PPI to assist in decision‐making regarding pleurodesis in patients with MPE.

### 5.1 Treatment Options

#### 5.1.1 Observation

For small pleural effusions and asymptomatic patients, observation is an option. However, if the effusion progresses or symptoms develop, further intervention is indicated.

#### 5.1.2 Thoracocentesis

Therapeutic thoracocentesis is the first‐line treatment for symptomatic MPE and is typically performed using a needle or catheter under ultrasound guidance. It helps determine the symptomatic response to drainage, the lung's ability to fully re‐expand, and the subsequent rate of fluid reaccumulation, all of which inform future definitive treatments [57].

We typically limit fluid removal to 1.5 L due to concerns that removing larger volumes may increase the risk of serious procedure‐related complications, particularly re‐expansion pulmonary edema. Although large‐volume drainage is often necessary, removing smaller volumes can provide symptom relief in patients with underlying lung disease. Careful monitoring of chest discomfort and cough during the procedure is advised to guide the amount of fluid removed and to assess whether the underlying lung is expandable or not [58].

#### 5.1.3 Chest Tube Drainage With Water Seal

Chest tube or small‐bore catheter drainage is an alternative to large‐volume thoracentesis and can be performed in patients who require slow fluid removal over hours or days for symptom relief. We generally prefer thin 14Fr drains, such as the pigtail catheter.

#### 5.1.4 Sclerosing Agent

The sclerosing agent of choice is talc. Besides being affordable and widely available, talc has a reported success rate of 60%–90% in preventing recurrence within 30 days after pleurodesis.

In the absence of talc, our alternative sclerosing agent is povidone–iodine (PVPI). Observational studies and small randomized trials report that PVPI achieved successful pleurodesis in 86%–96% of patients, with no significant difference in efficacy compared to talc [58].

The two most commonly used pleurodesis methods are:

Thoracostomy with “talc slurry”—Administration of talc via an intercostal chest tube in suspension form (powder mixed with saline).

Dose: 5 g of talc diluted in 50–100 mL of 0.9% saline or 20 mL of PVPI diluted in 80 mL of 0.9% saline (20% solution).

VATS “talc poudrage”—Direct talc powder application to the pleural surface during video‐assisted thoracoscopic surgery (VATS).

Dose: 5 g of talc applied directly over the entire pleural surface or 20 mL of PVPI diluted in 80 mL of 0.9% saline.

In general, clamping of the chest tube for 1–2 h after pleurodesis is recommended, with tube removal once drainage is < 150 mL/day [59, 60].

#### 5.1.5 Pleurodesis Indication

Pleurodesis should be reserved for patients with adequate lung re‐expansion, as this is the primary predictor of its success. A minimum of 50% re‐expansion is suggested as a criterion for indicating pleurodesis.

#### 5.1.6 Trapped Lung

Treatment options for this population are limited, with indwelling pleural catheters (IPC) being the preferred intervention. Ultrasound guidance can assist in placing the IPC in the area with the largest fluid septation to maximize symptom relief.

IPCs are thin, long‐term silicone chest drains that are tunneled under the skin, allowing patients to self‐manage pleural fluid drainage at home, avoiding hospitalization.

Pulmonary decortication and pleurectomy are not recommended in these cases due to high morbidity and low resolution rates [1].

The Eloesser pleurostomy should also not be routinely indicated, as it may have an increased risk of infection compared to less invasive methods, such as IPCs [61]. The infection rates of a MPE are associated with other factors, such as pleural procedures (e.g., repeated thoracentesis, IPC use, surgeries) and the development of pneumonia. However, isolated and spontaneous infection rates are minimal [1, 61]. The incidence of infection in patients with IPCs for MPE is relatively low.

#### 5.1.7 Pleurodesis Without Diagnostic Confirmation

Performing pleurodesis in cases of suspected MPE without a confirmed histological diagnosis should be approached with caution. According to the guidelines of the American College of Chest Physicians, in patients with suspected MPE and no confirmation of stage IV disease, thoracoscopy is recommended over tunneled pleural catheters due to its dual diagnostic and therapeutic benefits [62, 63].

Thoracoscopy allows direct inspection of the pleural cavity and the collection of biopsies, which can confirm the histological type and guide subsequent treatment.

Pleurodesis is generally reserved for cases where malignancy is confirmed, and the lung is re‐expandable, as its effectiveness depends on the lung's ability to obliterate the pleural space [62, 64]. Additionally, pleurodesis may not be appropriate when malignancy is suspected but not confirmed, as obtaining a histological diagnosis is crucial for proper therapeutic planning and to avoid unnecessary or inappropriate interventions.

Therefore, in cases of suspected malignancy without a histological diagnosis, the priority should be diagnostic confirmation, preferably through thoracoscopy, before considering pleurodesis as a therapeutic option.

**Figure 1:**
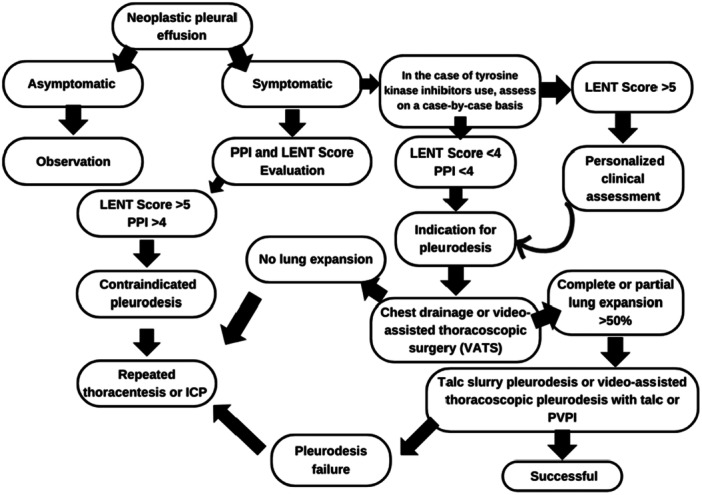
Flowchart for the use of survival scores and indication for pleurodesis.

After diagnosing MPE in symptomatic patients, we calculate survival scores to assess the indication for pleurodesis (Figure 1).

Based on survival scores, we created the following flowchart:

## 6 Complications

Pleurodesis in patients with low performance status may be associated with several complications, especially due to the fragility of these patients [65]. All patients with a low estimated survival based on pleural effusion survival scores have a low performance status. Patients with a KPS score ≥ 70 had a median survival of 395 days, whereas those with a KPS score ≤ 30 had a median survival of only 34 days [66]. Patients with a performance status below 50, according to our study, will not benefit from pleurodesis and may have an increased risk of complications associated with the procedure.

Studies indicate that pleurodesis can trigger a significant systemic inflammatory response, leading to adverse events such as pain, anemia, and elevated inflammatory markers, including CRP [65]. Furthermore, early and late mortality after pleurodesis may be influenced by factors such as ECOG performance status, where a status of 2–3 is associated with higher in‐hospital and 3‐month mortality [67]. The presence of multiple metastatic sites and low preoperative serum albumin levels are also risk factors for complications and mortality [67].

Another potential risk is the development of interstitial lung disease after pleurodesis, especially in elderly patients or those undergoing treatment with epidermal growth factor receptor tyrosine kinase inhibitors (EGFR‐TKIs) [68]. Therefore, the decision to perform pleurodesis in patients with low performance status should be carefully considered, taking into account the risks of complications and life expectancy, which can be assessed using prognostic tools such as the LENT score, with or without additional survival scores.

As we engage in the process of shared medical decision‐making with patients who have recurrent symptomatic MPE, advanced disease, and limited life expectancy, other patient‐related factors should be considered, favoring less invasive approaches to palliate symptoms and optimize quality of life [69]. A retrospective study by Pilling et al. [70] reviewed preoperative data from 278 patients admitted to a surgical unit for the evaluation of MPE treatment, most commonly talc pleurodesis via thoracoscopy. The authors found that leukocytosis, hypoxemia, and hypoalbuminemia were associated with poor prognosis. Patients presenting all three factors had a median survival of 42 versus 702 days (*p* < 0.00001). This finding also suggests that invasive interventions should be approached with considerable caution in patients with these characteristics [70].

Based on our review and our suggested approach, we will not recommend pleurodesis in patients with low survival expectancy and low performance status due to the risk of increasing the chance of complications.

### 6.1 Pharmacological Approach

**Figure 2:**
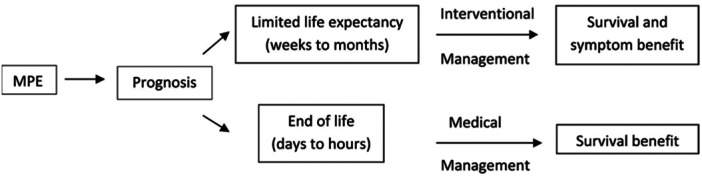
Approaches for the indication of pharmacological treatment of NPD. **Source:** Adapted from Beyea et al. [69].

Invasive procedures may be appropriate in outpatient settings or for patients with good performance status, but they may not be suitable when a patient is in the final days or weeks of a terminal illness (Figure 2). The pharmacological approach to managing MPE in this patient population should be tailored based on prognosis and therapeutic goals [69].

The primary pharmacological approaches include opioids, benzodiazepines, and supplemental oxygen, while nonpharmacological interventions involve modalities such as relaxation techniques, respiratory physiotherapy, and cognitive‐behavioral therapy [69].

## 7 Conclusion

The use of survival scores such as the PPI and LENT score are valuable tools for predicting survival in neoplastic pleural effusion and guiding decisions such as pleurodesis. It is important to consider adapting these scores to the specific characteristics of the population and the patient to maximize their clinical utility. Based on our review and our suggested approach, we will not recommend pleurodesis in patients with low survival expectancy and low performance status due to the risk of increasing the chance of complications.

## Conflicts of Interest

The authors declare no conflicts of interest.

## Synopsis

This article reports the importance of evaluating survival scores for the indication of pleurodesis. Based on our review and our suggested approach, we will not recommend pleurodesis in patients with low survival expectancy and low performance status due to the risk of increasing the chance of complications.

## Data Availability

The data that support the findings of this study are available from the corresponding author upon reasonable request.
